# Use of Stokes’ theorem for plasma confinement

**DOI:** 10.1098/rsta.2019.0519

**Published:** 2020-06-08

**Authors:** R. S. MacKay

**Affiliations:** Mathematics Institute, University of Warwick, Coventry CV4 7AL, UK

**Keywords:** Stokes’ theorem, magnetic fields, plasma confinement

## Abstract

Stokes’ theorem, in its original form and Cartan’s generalization, is crucial for designing magnetic fields to confine plasma (ionized gas). The paper illustrates its use, in particular to address the question whether quasi-symmetric fields, those for which guiding-centre motion is integrable, can be made with little or no toroidal current.

This article is part of the theme issue ‘Stokes at 200 (Part 1)’.

## 1. Introduction

In its original form, Stokes’ theorem says that for a three-dimensional vector field *B* and any surface *S*,

1.1 $$\int_{S}\text{curl}\;B\cdot \text{d}S=\int_{\partial S}B\cdot \text{d}l,$$

where ∂*S* is the boundary of *S*.

The attribution of the theorem to Stokes is questionable [1]. It was communicated by Thomson (future Lord Kelvin) to Stokes in 1850 and then set as an examination question by Stokes in 1854. Nevertheless, it fits with Stokes’ lines of thought and he may well have known the theorem before without enunciating it.

The paper begins by giving two immediate applications of Stokes’ theorem to magnetic fields. It then goes on to address the question whether a magnetic field with little or no toroidal current can be made in which guiding-centre motion is integrable.

The analysis of this question makes frequent use of Cartan’s generalization of Stokes’ theorem:

1.2 $$\int_{V}\text{d}\omega =\int_{\partial V}\omega ,$$

for any differential *k*-form *ω* and (*k* + 1)-dimensional submanifold *V* with boundary ∂*V*. The concepts of differential form and exterior derivative *d* will be reviewed, but it is interesting to note that at least differential 1-forms were familiar to Stokes. In his first publication [2], largely on irrotational flow, he does not say that the velocity field *u* is locally a gradient; he writes that ‘*u* d*x* + *v* d*y* + *w* d*z* is an exact differential’, meaning that this 1-form is *d* of some 0-form (function).

## 2. Immediate applications to magnetic fields

The goal of magnetically confined fusion reactor research is to make a magnetic field that confines deuterium and tritium nuclei long enough that they fuse. A magnetic field is a three-dimensional vector field *B* with div *B* = 0. Its effect on a particle of charge *e* is the Lorentz force *e*(*v* × *B*), where *v* is its velocity.

The design on which most of the effort and money has been invested so far is the ‘tokamak’. This is characterized by a close to axisymmetric magnetic field with a relatively strong toroidal current in the plasma. ‘Toroidal’ means in the direction of the axisymmetry.

The reason for the toroidal current is that without it, charged particles would drift vertically (up or down depending on the sign of charge), as will be explained in the next section. The opposite signs of vertical drift for opposite signs of charge would create a vertical electrostatic field, which one might think would limit the vertical drifts, but actually leads to both signs of charge drifting out radially. Even a small toroidal current is inadequate for confinement in an axisymmetric device because the particles would make large excursions, taking them out of the device.

The toroidal current makes the magnetic field wind round the plasma, because of Stokes’ theorem. Namely, the current density *J* is curl *B* (in units where the magnetic permeability *μ*_0_ = 1), so the current *I*(*D*) through a poloidal disc satisfies

2.1 $$I(D)=\int_{D}J\cdot \text{d}S=\int_{\partial D}B\cdot \text{d}l.$$

‘Poloidal’ means in a plane perpendicular to the direction of axisymmetry. Thus if there is a net toroidal current then the magnetic field has a net poloidal component.

By axisymmetry and volume preservation, the magnetic field lines lie on axisymmetric surfaces called flux surfaces, and because of the toroidal current these surfaces are closed, in fact tori. Furthermore, the magnetic field line flow on them is conjugate to a rotation. For flux surfaces with irrational winding, the time-averaged first-order drift of a circulating charged particle across flux surfaces is zero.

Next, to drive a steady toroidal current against plasma resistivity requires^1^ a steady toroidal electric field *E*. Yet Faraday’s equation is

2.2 $$\text{curl}\;E=-\frac{\partial B}{\partial t}.$$

So taking a toroidal loop *γ*, we obtain

2.3 $$\int_{\gamma }E\cdot \text{d}l=-\frac{\partial }{\partial t}\int_{D}B\cdot \text{d}S,$$

by Stokes’ theorem again, where *D* is a disc spanning *γ*. Thus the magnetic flux through *D* has to increase linearly and so indefinitely. This is impossible to achieve, so tokamaks have to operate in pulsed mode, with the magnetic flux through the centre being increased linearly for a while and then stopped.

Furthermore, toroidal current promotes instabilities. These do not necessarily saturate at small amplitude but may develop into what are known as ‘disruptions’ in which the plasma flips around wildly and may touch the wall of the container resulting in major damage.

It would be better to find a design that does not have much toroidal current. That requires departure from axisymmetry. We can nevertheless stay within the same topological class of configurations, which continue to have a toroidal direction and poloidal sections. Then Stokes’ theorem, as above, says that to minimize toroidal current we should make $\int_{\eta }B\cdot \text{d}l$ small around each poloidal loop *η*.

A beautiful idea of Spitzer is that winding of the magnetic field around the plasma, necessary to make the drifts average to zero, can be achieved without toroidal current by making the plasma twist non-axisymmetrically, a design he called ‘stellarator’. This was subsequently understood by [4] as a manifestation of a geometric phase.

The goal of stellarator research is to design a magnetic field that confines charged particles and requires no or little toroidal current. As we have seen that this can not be achieved with an axisymmetric magnetic field, the analysis and design is harder. To get started we need to review the concepts of guiding-centre motion and flux surfaces.

## 3. Guiding-centre motion

In a uniform magnetic field, the motion of a charged particle is at constant rotation rate along a helix around a field line. The rotation rate is the *gyrofrequency* *Ω* = *e*|*B*|/*m*. The radius of the helix, called the gyro-radius, is the perpendicular momentum divided by *e*|*B*|. If the field varies slowly on the scales of the gyro-radius and $v_{∥}/\Omega$, where $v_{∥}$ is the parallel velocity, then it is convenient to transform from the particle position *q* and perpendicular velocity *v*_⊥_ to a *guiding-centre position* *X* and *gyro-radius vector* *ρ* perpendicular to *B*(*X*) defined implicitly by

3.1 $$v_{⊥}=\frac{e}{m}B(X)\times \rho$$

and

3.2 q=X+ρ.

It follows from the approximate gyro-rotation symmetry that the *magnetic moment* *μ* = (*m* |*v*_⊥_|^2^)/(2|*B*|) is an adiabatic invariant. Thus the particle position *q* remains within about $\sqrt{2\mu /e\Omega }$ of *X* for long times.

At zeroth order in the ratios of |*ρ*| and $v_{∥}/\Omega$ to the length scale of variation of *B*, the guiding-centre *X* moves along a fieldline, conserving the energy $H=\frac{1}{2}mv_{∥}^{2}+\mu |B(X)|$. Thus it circulates along the field in a constant direction if the value *E* of the energy is large enough. Otherwise, it bounces at places where |*B*(*X*)| = *E*/*μ*.

At first order, *X* drifts across the field lines according to

3.3 $$\dot{X}=v_{∥}b+\frac{mv_{∥}^{2}}{e|B|}c_{⊥}+\frac{\mu }{e|B|}b\times \nabla |B|$$

and

3.4 $${\dot{v}}_{∥}=-\frac{\mu }{m}b\cdot \nabla |B|,$$

where *b* = *B*/|*B*| and *c* = curl *b* (*c*_⊥_ can be written as b×(b⋅∇)b). In (3.3) the first term is the zeroth order motion along the field, the second is a drift due to curvature of the fieldlines, and the third is a drift due to gradient of the field strength. The latter is analogous to Stokes’ drift of particles in water waves, where the radius of the approximately circular motion decreases with depth, producing an average horizontal drift. Here the radius of the approximately circular motion decreases with increasing field strength.

As an example, if *B* is axisymmetric and toroidal then the drifts are both vertical, as claimed in the previous section.

## 4. Flux surfaces

To confine the circulating guiding centres, we see from the previous section that it is necessary at least to confine the fieldlines. A good way to achieve this is to design the field to have flux surfaces, which in contrast to the axisymmetric case is not automatic for a general three-dimensional magnetic field.

We say a magnetic field *B* has flux surfaces if there is a function *ψ* with derivative non-zero almost everywhere such that B⋅∇ψ=0, called a *flux function*. Then the regular^2^ components of level sets of *ψ* form surfaces and the field lines starting on such a surface stay on it. If *B* ≠ 0 everywhere on a bounded regular component of a level set of *ψ*, called a *flux surface*, then it is a torus.

Actually, one usually wants a bit more, namely that there is a divergence-free vector field *u* such that u×B=∇ψ and *u*, *B* are linearly independent (equivalently ∇ψ≠0) almost everywhere. It follows then that the magnetic field on a bounded regular component of a level set of *ψ* is conjugate to a constant vector field. The torus bounds a solid torus. On the torus, one can identify a poloidal class of non-contractible loops, which can be contracted to a point in the solid torus, and a toroidal class of non-contractible loops, which cut a poloidal loop precisely once. The long-time ratio of the number of turns a field line makes in the poloidal direction to the toroidal direction is called the *winding number*, or rotational transform, for the torus, and denoted by ι.

Existence of flux surfaces is automatic if the plasma is in *magnetohydrostatic equilibrium*,^3^ i.e. J×B=∇p, with non-zero pressure gradient almost everywhere, because *J* serves as the field *u* and *p* serves as a flux function. Nevertheless, *p* fails to have non-zero gradient outside the plasma and has zero gradient at every rational surface unless an associated Fourier component of the field strength is zero [5,6]. So existence of flux surfaces is not quite as automatic as one might have hoped.

If a magnetic field has flux surfaces then the rate of change of the flux function for first-order guiding centre motion (3.3) averaged along the zeroth order guiding-centre motion along a field line with irrational winding ratio is zero (e.g. [7]). This can fail, however, for circulating particles with rational winding ratio and for bouncing particles. One way forward, called *omnigenity*, is to design the field so that the average drift rate is zero for all zeroth order motions. We prefer, however, to ask for a stronger condition, namely that first-order guiding-centre motion be integrable. This is called *quasi-symmetry*.

To formulate quasi-symmetry in detail (and integrability), I use a Hamiltonian formulation of guiding-centre motion and choose to use the language of differential forms, so first give a short tutorial on that (for a longer one, see [8]).

## 5. Differential forms

On a manifold of dimension *n*, for integers *k* = 0, …*n*, a *k*-*form* is a *k*-linear map from *k*-tuples of tangent vectors at a point to the reals. For example, given a vector field *B* and a Riemannian metric, we define the 1-form $B^{♭}$ by $B^{♭}(v)=B\cdot v$ for all vectors *v*, where *B* · *v* denotes the inner product of the vectors using the Riemannian metric. In three dimensions, we can also make a 2-form *β* from *B* by *β*(*v*, *w*) = *B* · (*v* × *w*), where the cross product is also defined with the aid of the Riemannian metric; it represents the flux of *B* through the infinitesimal parallelogram spanned by *v* and *w*. Also in three dimensions, we can define a 3-form *Ω* by *Ω*(*u*, *v*, *w*) = *u* · (*v* × *w*), representing the signed volume of the parallelopiped spanned by *u*, *v* and *w*. A 0-form takes no tangent vectors as arguments but may still depend on the point on the manifold, so is just a scalar function on the manifold, e.g. |*B*|.

A *k*-form can be integrated over any smooth *k*-submanifold. Simply divide the submanifold into little parallelopipeds, sum up the values of the *k*-form on them, and take the limit as the mesh size goes to zero.

An important operator on differential forms is the exterior derivative *d*. It takes a *k*-form (with *k* < *n*) to a (*k* + 1)-form. For *k*-form *ω*, d*ω*(*ξ*_1_, …*ξ*_*k*+1_) is defined to be the limit as ε → 0 of $\varepsilon^{-(k+1)}\int_{\partial V_{\varepsilon }}\omega$, where *V*_ε_ is the parallelopiped spanned by ε*ξ*_1_, …ε*ξ*_*k*+1_ in a local coordinate system and ∂*V*_ε_ is its boundary (*d* of an *n*-form is defined to be 0).

From this definition follows Cartan’s generalization of Stokes’ theorem:

5.1 $$\int_{V}\text{d}\omega =\int_{\partial V}\omega$$

for any (*k* + 1)-submanifold *V*.

Cartan’s generalization includes Stokes’ theorem, as follows. For a vector field *B* in three dimensions, *J* = curl *B* can be defined to be the vector field such that $i_{J}\Omega =\text{d}B^{♭}$, where *i*_*J*_ is the operator on differential forms that inserts *J* as the first argument (on a 0-form it is defined to give 0). So apply Cartan’s generalization to $\omega =B^{♭}$ to obtain Stokes’ theorem.

It also includes the Gauss–Ostrogradskii divergence theorem $\int_{V}\text{div}\;B\;\text{d}V=\int_{\partial V}B\cdot \text{d}S$ for a three-dimensional volume *V*, because take *ω* = *i*_*B*_*Ω* and use the definition of div *B* as the scalar function such that *di*_*B*_*Ω* = (div *B*)*Ω*. Such a function exists because the space of top-dimensional forms at a point is one-dimensional and *Ω* is assumed to be non-degenerate, so *di*_*B*_*Ω* is some multiple of *Ω*. Note in particular that div *B* = 0 iff d*β* = 0 (where *β* = *i*_*B*_*Ω*). We say a form *ω* for which d*ω* = 0 is *closed*.

A form *ω* is called *non-degenerate* if *i*_*v*_*ω* = 0 implies the vector field *v* is zero.

Given a vector field *v*, the *Lie derivative* *L*_*v*_ describes how a differential form evolves along *v* (it can be applied to other tensors too). More precisely, if *ω* is a *k*-form and *S*(*t*) is a *k*-submanifold that flows with *v* then

5.2 $$\frac{\text{d}}{\text{d}t}\int_{S(t)}\omega =\int_{S(t)}L_{v}\omega .$$

On a 0-form (scalar function) *f*, *L*_*v*_*f* is simply the directional derivative d*f*(*v*). On differential forms more generally, it has the formula

5.3 $$L_{v}=i_{v}d+di_{v}.$$

## 6. Hamiltonian systems

Differential forms allow a neat formulation of Hamiltonian dynamics that provides a useful generalization of the standard formulation in canonical coordinates.

In this generalization, a Hamiltonian system is specified by a scalar function *H*, called the *Hamiltonian*, and a symplectic form *ω* on the state-space. A *symplectic form* is a closed non-degenerate 2-form. Existence of a symplectic form imposes constraints on the state space. In particular it must have even dimension. Half the dimension is called the *number of degrees of freedom* (d.f.). The dynamics is given by the vector field *V* such that

6.1 $$i_{V}\omega =\text{d}H.$$

By non-degeneracy of *ω*, there exists such a *V* and it is unique.

The key example for present purposes is first-order guiding-centre motion. The state space is the four-dimensional set of possible guiding-centre positions *X* and parallel velocities $v_{∥}$; we will denote the latter by just *v*. We already identified the Hamiltonian function

6.2 $$H=\frac{1}{2}mv^{2}+\mu |B(X)|.$$

The symplectic form is

6.3 $$\omega =-e\beta -md(vb^{♭}),$$

which is closed and can be checked to be non-degenerate wherever ${\tilde{B}}_{∥}$, to be defined below, is non-zero. Solving (6.1) for $V=(\dot{X},\dot{v})$ yields

6.4 $$\dot{X}=\left(v\tilde{B}(X)+\frac{\mu }{e}b\times \nabla |B|\right)/{\tilde{B}}_{∥}$$

and

6.5 $$\dot{v}=-\frac{\mu }{m}\frac{\tilde{B}}{{\tilde{B}}_{∥}}\cdot \nabla |B|,$$

where $\tilde{B}=B+(m/e)vc$ (recall *c* = curl *b*) and ${\tilde{B}}_{∥}=\tilde{B}\cdot b$. This agrees to first order with the previous equations (3.3) and (3.4) but has the advantage of Hamiltonian structure. It was derived by Littlejohn [9].

An important result for Hamiltonian systems is that a continuous symmetry implies a conserved quantity (the Hamiltonian version of Noether’s theorem). A vector field *U* is a *continuous symmetry* of Hamiltonian system (*H*, *ω*) if *L*_*U*_ *H* = 0 and *L*_*U*_*ω* = 0. It follows that there is locally a function *K* which is conserved by the Hamiltonian vector field *V*, i.e. *i*_*V*_ d*K* = 0. This can be proved using Cartan’s form of Stokes’ theorem, applied to the 1-form *i*_*U*_*ω*, as follows. *L*_*U*_*ω* = 0 and d*ω* = 0 imply *di*_*U*_*ω* = 0. So for a curve *γ* from a point *x*_0_ to a point *x*, $\int_{\gamma }i_{U}\omega$ does not change if *γ* is continuously deformed to another path connecting the same two points. In a neighbourhood of *x*_0_, we define *K*(*x*) to be the value of this integral. Then d*K* = *i*_*U*_*ω*, so *i*_*V*_ d*K* = *i*_*V*_*i*_*U*_*ω* = −*i*_*U*_ d*H* = 0, from *L*_*U*_*H* = 0.

The local function *K* fails to be globally defined if there is a non-contractible loop *γ* around which $\int_{\gamma }i_{U}\omega \neq 0$. Otherwise *K* is global. We say a 2 d.f. system (*H*, *ω*) is *integrable* if it has a globally defined conserved quantity *K* such that d*H* and d*K* are independent almost everywhere.

## 7. Quasi-symmetry

We say a magnetic field *B* is *quasi-symmetric* if there is a three-dimensional vector field *u* such that *U* = (*u*, 0) makes guiding-centre motion integrable. We have obtained the following result [10].

**Theorem 7.1.**

(*u*, 0) *is a continuous symmetry of guiding-centre motion iff*

*L*_*u*_|*B*| = 0 (*equivalently*, u⋅∇|B|=0)
*L*_*u*_*β* = 0 (*equivalently, curl* (*B* × *u*) = 0)
$L_{u}B^{♭}=0$ (*equivalently*, u×J=∇(u⋅B)).

*B is quasi-symmetric iff there is such a vector field u, i*_*u*_*β* = d*ψ* (*equivalently*, B×u=∇ψ) *for some global function ψ, and u*, *B are independent* (*equivalently dψ* ≠ 0) *almost everywhere*.

**Sketch of proof.**

Firstly *L*_*u*_*H* = 0 with *H* in (6.2) iff *L*_*u*_|*B*| = 0. Secondly, *L*_*u*_*ω* = 0 with *ω* in (6.3) can be shown to be equivalent to *L*_*u*_*β* = 0 and $L_{u}b^{♭}=0$. Then

7.1 $$L_{u}B^{♭}=L_{u}(|B|b^{♭})=(L_{u}|B|)b^{♭}+|B|L_{u}b^{♭}.$$

Next, because div *B* = 0 then *L*_*u*_*β* = 0 iff *i*_*u*_*β* = d*ψ* for some local function *ψ*. This is another application of Stokes’ theorem, just as in the proof of Noether’s theorem above. The local conserved quantity for guiding centre motion comes out to be

7.2 K=−eψ−mvu⋅b,

so *K* is global iff *ψ* is global. Lastly, d*H* and d*K* are independent at (*X*, *v*) iff *r* d*H* + *s* d*K* = 0 there for r,s∈R implies *r* = *s* = 0. Now

7.3 r dH+s dK=r(mv dv+μ d|B|)−s(e dψ+mu⋅b dv+mv d(u⋅b)).

If this is zero and (*r*, *s*) ≠ (0, 0) then

7.4 u⋅bμ d|B|−v(e dψ+mv d(u⋅b))=0,

but this is quadratic in *v*, so if d*ψ* ≠ 0 at *X* it is zero for at most two values of *v* (typically for none, because the coefficients are 1-forms in three dimensions). The fields *u*, *B* are independent at *X* iff d*ψ* ≠ 0 at *X*, so *u*, *B* are independent at almost every *X* iff d*H*, d*K* are independent at almost every (*X*, *v*). ▪

Under the global condition for *ψ* and independence of *u*, *B*, the bounded components of level sets of *ψ* are flux surfaces. So using conservation of *K* (7.2), a guiding centre remains within order *v* of its initial flux surface, with variation of *ψ* given by the change in (*m*/*e*) *v u* · *b*. The variation in *v* is given by conservation of *H*. Thus quasi-symmetry produces excellent single-particle confinement.

The question remains whether quasi-symmetry can be realized with zero toroidal current. Axisymmetry is an example of quasi-symmetry, but, as we have seen, one requires toroidal current to make bounded flux surfaces for an axisymmetric field.

We have obtained strong restrictions on quasi-symmetries [10], beyond the existence of flux surfaces. Notably, div *u* = 0 and *u* commutes with *B* and with *J* (if the field is also magnetohydrostatic then all three commute). The quantity *u* · *B* is constant along *u* and under mild conditions every orbit of *u* is periodic. Four independent combinations of the six components of *L*_*u*_*g* are zero, where *g* denotes the Euclidean metric. A homogeneous quintic in the components of the derivative of *u* is zero.

It may be that there are no quasi-symmetries apart from axisymmetries. This would hold if all components of *L*_*u*_*g* are zero.

Or it may be that there are ‘Kovalevskaya examples’. The latter term refers to the theory of tops—rigid bodies freely rotating about a fixed point in a uniform gravitational field. If the fixed point is at the centre of mass then the motion is integrable (Euler). If the inertia tensor of the top has rotation symmetry about an axis through the fixed point and the centre of mass is on the axis then the motion is integrable (Lagrange). Kovalevskaya discovered another integrable class of tops: the inertia tensor about the fixed point has rotation symmetry about an axis, the moments of inertia are in the ratio 1 : 2 : 2 and the centre of mass is in the plane through the fixed point perpendicular to the symmetry axis [11]. If we can find non-axisymmetric quasi-symmetric magnetic fields we would then have to examine their toroidal current $\int_{\eta }B^{♭}$ over poloidal loops *η*. Watch this space!

## 8. Conclusion

Stokes’ theorem is a powerful tool for plasma physics. We are using it to design magnetic confinement devices.

## Footnotes

1 Actually, this is an oversimplification, because currents can also be driven by electromagnetic waves, neutral beams and pressure gradients, notably the ‘bootstrap’ current [3]. Solving the dependence on magnetic induction to start the toroidal current and maintaining it, however, remains a significant challenge for tokamaks.

2 A component of a level set of *ψ* is ‘regular’ if d*ψ* ≠ 0 everywhere on it.

3 This is not a standard term, but is the special case of magnetohydrodynamic equilibrium with isotropic pressure and no mean flow.

## Data accessibility

This article has no additional data.
